# Examining Possible Determinants of Kinesiology Students’ Social Physique Anxiety: A Multiple Indicator Multiple Independent Cause Model

**DOI:** 10.1177/00315125241301849

**Published:** 2024-11-21

**Authors:** Jianmin Guan, Ping Xiang, William Land, Alberto Cordova

**Affiliations:** 1 12346 Department of Kinesiology, University of Texas at San Antonio, San Antonio, TX, USA; 2 14736 Department of Kinesiology, Texas A&M University, College Station, TX, USA

**Keywords:** kinesiology, MIMIC model, perceived stress, social physique anxiety, BMI

## Abstract

In this study, we expanded on previous research by employing a Multiple Indicators, Multiple Independent Causes (MIMIC) model to investigate how gender, age, body mass index (BMI), and perceived stress may have collectively influenced social physique anxiety (SPA) among 593 college kinesiology students. Our MIMIC model revealed that perceived stress, gender, and BMI were significantly related to SPA scores, with perceived stress being the strongest factor. Moreover, the relationship between gender and kinesiology students’ SPA scores was consistent across different ages. These findings broaden the spectrum of the current nomological network regarding predictors of SPA. Based on these findings, future researchers should extend the current MIMIC model by including more predictor variables (e.g., depression, mood, & mental toughness) to gain additional and perhaps deeper insights. Additionally, we advocate for the adoption of the MIMIC model of statistical analysis in future SPA research.

## Introduction

Social physique anxiety (SPA) refers to an individual’s feelings and concerns about their physical appearance as assessed by others in real or hypothetical situations (Hart et al., 1989). SPA is important because it may impact the individual’s motives, attitudes, preferences, self-perceptions, perceived abilities, enjoyment, and engagement or avoidance of physical activity (Sabiston et al., 2014). Considerable research evidence has revealed that SPA experiences are related to adverse effects on people’s physical and mental well-being (Brunet & Sabiston, 2009; Diehl et al., 1998; Lantz et al., 1997; Manuel et al., 2017; Woodman & Steer, 2011; Zartaloudi et al., 2023). To prevent such adverse effects, researchers must delve into possible underlying determinants of SPA.

To date, research on personal SPA experiences has identified numerous correlates and possible determinants, such as age, gender, body mass index (BMI), types of physical activity, environmental factors, and social factors (for a review, see Sabiston et al., 2014). Although prior research has played a crucial role in enhancing our comprehension of SPA, there are notable gaps in it. First, existing SPA studies with college students have not addressed kinesiology students. Since kinesiology students focus academically and in their future professional pursuits on physical activities, psychomotor skills, and exercise science, they may personally encounter higher levels of social pressure to maintain a fit and healthy physique. For example, kinesiology students are frequently evaluated on their own physical abilities and fitness levels. This constant evaluation, associated with class performance expectations, may lead to their personal concerns about their own physical performances and their fears of not meeting the expectations of their professors or instructors. Additionally, the nature of the kinesiology field places a strong emphasis on having a “perfect” or “ideal” physique. This pressure to maintain a certain body image can create anxiety and pressure about body size, shape, and appearance among these students particularly. Lastly, within a kinesiology program, students often interact with peers who excel in physical fitness and athletic performance. This environment may prompt social comparisons in which students feel the need to measure up to their classmates, and this can also contribute to heightened SPA. To further advance SPA research, it would be beneficial for researchers to expand their investigations specifically to kinesiology students.

A second gap in contemporary SPA research pertains to statistical methods commonly used. Most prior research in this area has relied on traditional cross-sectional, multiple regression analysis, analyses of variance (ANOVAs), and multiple analyses of variance (MANOVAs) to identify significant correlated independent variables. There is a methodological drawback in the use of multiple regression analysis, as it assumes that the measurement of predictor variables in the model is free of errors. When this assumption is violated, multiple regression analysis may produce biased regression coefficients and lead to inaccurate conclusions. To address this limitation, a more robust statistical approach known as the Multiple Indicators, Multiple Independent Causes Model (MichiganMIC) has recently gained attention. The MIMIC model, also called a mixed model measurement, incorporates both effect indicators and causal indicators into its latent variable or factor (Kline, 2023). As such, the MIMIC model is a combination of a reflective model and a causal-formative model. One notable advantage of the MIMIC model is its ability to simultaneously estimate the relationships between covariables and latent variables, along with the factor loadings in the model. Unlike ANOVAs or MANOVAs, the MIMIC model can readily integrate a greater number of independent variables (i.e., covariates or predictors) into data analyses. Compared to multiple regression, the MIMIC model can not only calculate the measurement error of the latent variables, but it can also concurrently estimate the unknown coefficients among the latent variables, giving it greater power and flexibility (Chang et al., 2020). In this study, we employed a MIMIC model to investigate the associations between predictor variables and SPA levels. We anticipated that utilizing the MIMIC model would make a distinctive contribution to existing SPA research.

A third gap in existing SPA research involves sparse attention to exploring the interactive impacts of participants’ gender and age on SPA, particularly when considering age as a continuous variable. In the existing literature, gender and age have emerged as potentially the most valuable predictors of SPA. Typically, females tend to encounter elevated levels of SPA in comparison to males. (Chu et al., 2008; Eklund et al., 1997; Ersoz, 2016; Haase & Prapavessis, 1998; Hagger & Stevenson, 2010; Mack et al., 2007; Silva et al., 2022; Swami et al., 2021; Zartaloudi et al., 2023). Additionally, being of a younger age has been linked to experiencing higher levels of SPA (Hagger & Stevenson, 2010; Hausenblas & Martin, 2002; Silva et al., 2022; Thompson & Chad, 2002). Although gender differences and age variations have been consistently associated with SPA, most prior investigators have concentrated on these variables individually as main effects on SPA in analyses of variance. To our knowledge, the sole study examining the interactive impacts of age by gender on SPA was conducted by Hagger and Stevenson (2010). These authors examined the interactive effects of gender and age on SPA through six distinct age groups (i.e., 11-12, 13-14, 15-16, 17-18, 19-20, and 21+ years old) of participants in the UK. Although there were significant interaction effects between age and gender on SPA, the only significant age group by gender difference was that 11-12-year-old females showed lower SPA values than all older female age groups except the 15-16-year-old group. Additionally, in all age groups except the 11-12-year-old group, females’ SPA scores were significantly higher than those of males. Notably, Hagger and Stevenson (2010) did not consider or control other potential factors or covariates, and, as noted earlier, no investigators have addressed age by gender interaction effects on SPA among U.S. college kinesiology students.

The fourth gap in SPA research concerns a sparsity of research differences between individuals with and without normal Body Mass Index (BMI) values. Many researchers have shown that SPA can be related to BMI (Ersöz et al., 2016; Hausenblas & Fallon, 2002; Mack et al., 2007; Russell, 2002; Sabiston et al., 2005; Thogersen-Ntoumani & Ntoumanis, 2007; Zartaloudi et al., 2023). For example, Mack et al. (2007) reported that BMI positively predicted SPA in a regression analysis with high school students, with higher BMI associated with higher SPA scores. Hausenblas and Fallon (2002) examined the relationships between body image, exercise behavior, BMI, and primary exercise dependence symptoms in physically active college students. They showed that BMI was most strongly and positively linked with SPA in females, whereas exercise behavior was most strongly and negatively associated with SPA in males. However, most prior researchers who focused on relationships between BMI and SPA failed to address whether these relationships depended on normal or abnormal (underweight, overweight, and obesity) BMI values. Again, this gap is particularly notable among kinesiology students.

The last SPA research gap pertains to a lack of attention to variant aspects or underlying mechanisms of SPA. According to Sabiston et al. (2014), research on predictors of SPA has mainly focused on participants’ personal characteristics, types of physical activity, environmental factors, social factors, and self-perceptions. To enhance our understanding of underlying SPA mechanisms, the existing nomological SPA network should be expanded by exploring more potential SPA predictors or antecedents.

In this study, we hoped to fill existing research gaps by also examining associations between kinesiology students’ perceived stress and their SPA scores. Perceived stress refers to the individual’s feelings or thoughts regarding current stress levels (Phillips, 2013). We posited that SPA would be positively linked to perceived stress, given the intertwining of stress and anxiety across various facets of life. For example, if an individual feels stress about an ideal body image due to societal norms, this perceived stress could be associated with the exacerbation of SPA. For instance, if an individual experiences stress related to how they perceive their own physique and believes that others are negatively judging them based on their physical appearance, they may experience heightened levels of SPA. Given that perceived physical appearance or body image is a component of both perceived stress and SPA, we expected that one’s perceived stress might be positively related to SPA levels, particularly in stressful social interactions, such as feeling judged or stigmatized, based on physical appearance. Our aim in this study was to employ a MIMIC model to test the following presumed predictors of SPA: gender, age, BMI, and perceived stress among college students majoring in kinesiology. Drawing from the existing research literature, we specifically hypothesized that female and younger students would exhibit higher SPA levels relative to males and older students. Additionally, we expected that the influence of gender on SPA would be consistent across different ages. Lastly, we hypothesized that students with normal BMI values or lower perceived stress would report lower SPA levels compared to those with abnormal BMI values or higher perceived stress.

## Method

### Ethical Considerations

Prior to data collection from any participants, we obtained approval for this research protocol from our university Institutional Review Board (IRB). Additionally, all participants gave their voluntary informed consent. To ensure the autonomy of the participants’ responses, we assured the students that their responses would be confidential, and that their participation was voluntary and anonymous. Lastly, we informed participants that the data would be used for research purposes and only disseminated in aggregate form, with no ties to individual participants’ identities.

### Participants

We collected data from 625 college kinesiology students aged 18–24 years. To ensure the accuracy of data analyses, we excluded case data with missing values for gender, age, and/or BMI (height or weight). Additionally, we removed two case outliers identified through Mahalanobis d-squared values. Thus, data analyses were based on 593 (245 male, 348 female) kinesiology students. These participants were recruited through convenience sampling from a university in the southern region of the United States, with the following ethnicities: Hispanic-American (63.7%), Caucasian (16.6%), African-American (13.2%), Asian-American (4.4%), and others (2.0%).

### Measures

#### Social Physique Anxiety Scale (SPAS)

Students’ SPA levels were measured using the previously validated 7-item single-factor SPAS (Motl & Conroy, 2000; Pacewicz et al., 2023). The SPAS consists of one positively worded item (i.e., “I am comfortable with how fit my body appears to others”) and six negatively worded items (e.g., “I wish I wasn’t so uptight about my physique/figure.). Respondents rated each item on a 5-point Likert-type scale, ranging from 1 (not true of me) to 5 (very true of me). To enhance the scale’s relevance to physical activity settings, we added the phrase “in physical activity settings” to all seven items. Furthermore, we adjusted one item by changing the wording from “Unattractive features of my physique/figure make me nervous in certain social settings” to “Unattractive features of my physique/figure make me nervous.” The modified SPAS demonstrated acceptable internal consistency and construct validity in this sample, as further elaborated in the Results section of this paper.

#### Perceived Stress Scale (PSS)

We employed the 10-item PSS scale validated by Cohen and Williamson (1988) to evaluate the frequency of individuals’ perceived stress experiences over the past month using a 5-point Likert scale (1 = never, 2 = almost never, 3 = sometimes, 4 = fairly often, and 5 = very often). This scale consists of two subscales: (i) perceived helplessness and (ii) perceived self-efficacy. The perceived helplessness subscale is comprised of six negatively worded items (e.g., “In the last month, how often have you felt nervous and stressed?”), while the perceived self-efficacy subscale includes four positively phrased items (e.g., “In the last month, how often have you felt that things were going your way?”). Based on data from within the current sample, Cronbach’s alpha coefficients for the perceived helplessness and perceived self-efficacy subscales were .84, and .71, respectively, indicating that the scores generated by the PSS were internally consistent and reliable. Confirmatory factor analysis (CFA) revealed that the construct validity of PSS was acceptable, with chi-square (*χ*^*2*^) = 146.41, *p* < .01; comparative fit index (CFI) = .94; goodness-of-fit (GFI) = .95; root mean square error of approximation (RMSEA) = .07. In this study, the total PSS score served as a covariate (i.e., predictor), calculated by reversing the scores of the four positively phrased items and then adding the scores for all ten items. Thus, individual scores on the PSS can range from 10 to 50 with a higher score denoting higher degree of perceived stress.

#### BMI

We calculated participants’ BMI values from their self-reported weight and height, using the formula: BMI = weight (kg)/height (m^2^). Based on the World Health Organization’s criteria (1998), we categorized BMI values into four groups: Underweight (<18.5), Normal (18.5-24.9), Overweight (25.0-29.9), and Obesity (30.0+). The groups were assigned the following codes: zero (normal, serving as the reference group), 1 (underweight), 2 (overweight), and 3 (obesity). Consequently, three dummy variables were established to compare underweight versus normal, overweight versus normal, and obesity versus normal, respectively. However, because only 12 kinesiology students were classified as underweight, we focused on just two comparisons: normal versus overweight and normal versus obesity.

### Data Collection and Statistical Analysis

We administered the combined questionnaire to participants during 16 regular class sessions. The completion time for the questionnaire was typically 20–25 minutes. We performed all statistical analyses using the IBM SPSS (version 29) and AMOS (version 29). We resolved missing data for SPAS and PSS through regression imputation. We calculated descriptive statistics, such as means and standard deviations, to portray participants’ characteristics and their SPA scores and covariate values. We employed a MIMIC model to investigate the association between students’ SPA levels and six potential predictors: gender (0 = male; 1 = female), age, interaction of age by gender, perceived stress, BMI normal versus overweight, and BMI normal versus obesity. To examine the possible interaction effects of gender across different ages, we created an interaction variable of age by gender within the MIMIC model. Prior to multiplication (gender by age), we first centered the continuous variable (age).

Given that the MIMIC model, a special form of Structural Equation Modeling, is based on an analysis of covariance structures, we assessed multivariate normality of the data distributions to ensure that this critical assumption was met. According to Bentler (2005) and Byrne (2016), Mardia’s normalized estimates surpassing 5.00 indicate the presence of multivariate nonnormality in the sample. Preliminary analyses revealed that Mardia’s normalized estimate for multivariate kurtosis was 4.63, signifying that the assumption of multivariate nonnormality was met.

The MIMIC model analysis involved two steps. In the first step, as noted in our description of the SAPS, we performed a CFA to assess a measurement model of the modified SPAS. To investigate whether variations in SPA scores between genders could be attributed to gender-related differences in the measurement properties of the modified SPAS, we conducted an additional multigroup analysis for the measurement model of the modified SPAS. In the second step, following confirmation of a good fit for the modified SPAS, we evaluated the hypothesized MIMIC model with covariates. We used a maximum likelihood estimation method to estimate the CFA and MIMIC models. We evaluated overall goodness-of-fit of the models with these data using multiple indices: CFI, GFI, and RMSEA. Typically, CFI and GFI values greater than .90 (Hu & Bentler, 1995), and a RMSEA value below .05 indicate an acceptable fit. Additionally, we considered RMSEA values between .05 and .08 as indicative of a marginal fit of the model (Browne & Cudeck, 1993). Lastly, given that the χ^2^-difference (Δχ^2^) test can be overly strict, we employed the CFI-difference (ΔCFI) test recommended by Cheung and Rensvold (2002) to assess the multigroup invariance for the modified SPAS measurement model. The threshold for ΔCFI for multigroup invariance should not exceed .01 (Cheung & Rensvold, 2002).

## Results

Table 1 presents the participants’ descriptive statistics in terms of their age, gender, perceived stress, BMI, overall SPA scores, and individual item scores of the modified SPAS. Compared to males, female students exhibited higher mean perceived stress scores, higher overall SPA scores, and higher individual SPA item scores. However, male students, on average, reported higher BMI values.

**Table 1.** Participant Descriptive Statistics, SPA Scores and Predictor Variable Scores.

			Gender			
	Full sample (*N* = 593)		Male (*n* = 245)		Female (*n* = 348)	
Variable	*M*	*SD*	*M*	*SD*	*M*	*SD*
Age	20.04	1.71	20.29	1.79	19.86	1.62
Perceived stress	30.25	6.55	28.12	6.30	31.74	6.31
BMI	25.93	5.11	27.16	4.88	25.07	5.09
SPA (overall)	3.29	.98	2.84	.93	3.61	.89
Item 1	2.81	1.19	2.45	1.11	3.07	1.17
Item 2	3.39	1.28	3.04	1.24	3.64	1.24
Item 3	3.39	1.40	2.80	1.35	3.81	1.28
Item 4	3.43	1.28	3.03	1.28	3.72	1.19
Item 5	3.12	1.21	2.87	1.14	3.29	1.22
Item 6	3.56	1.38	2.81	1.32	4.09	1.17
Item 7	3.35	1.29	2.90	1.27	3.67	1.21

CFA results showed that the modified 7-item SPAS model fit the data well (*χ*^2^ = 52.17, *p* < .001; CFI = .98; GFI = .98; RMSEA = .07). All seven items demonstrated significant factor loadings ranging from .64 to .80, signifying a robust association between the items and the SPA general factor. Additionally, Cronbach’s alpha coefficient was .88, indicating that the revised SPAS was internally consistent and reliable. Table 2 provides a summary of goodness-of-fit statistics for gender-related tests of multigroup invariance. Although the Δχ^2^ difference test provided evidence of partial gender invariance for the revised SPAS, the ΔCFI test showed that all of ΔCFI values were less than the .01 cutoff point suggested by Cheung and Rensvold (2002), indicating that the SPAS was completely invariant for males and females.

**Table 2.** Summary of Goodness-of-Fit Statistics for Gender-Related Tests of Multigroup Invariance.

Model description	Comparative model	χ^2^	df	Δχ^2^	Δdf	*p* value	CFI	ΔCFI
Unconstrained (Model A)	_	62.652	28	_	_	_	.978	_
Measurement weights (Model B)	Model B versus Model A	75.623	34	12.971	6	.043	.973	.005
Structural covariances (Model C)	Model C versus Model A	76.470	35	13.818	7	.055	.973	.005
Measurement residuals (Model D)	Model D versus Model A	85.959	42	23.307	14	.055	.971	.007

*Notes:* Δχ^2^ = Difference in Chi-square values between models; Δdf = Difference of degree of freedom between models; ΔCFA = Difference in CFI values between models.

**Figure 1.**
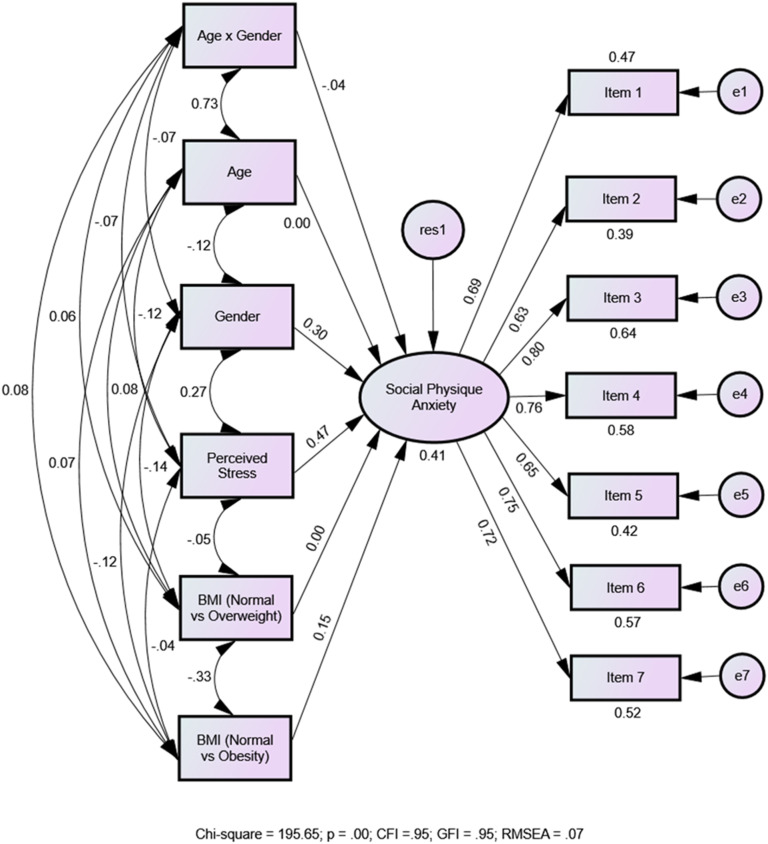
Standardized Results for the MIMIC Model in Predicting SPA from Gender, Age, Perceived Stress, BMI (Normal vs. Overweight) and BMI (Normal vs. Obesity).

The MIMIC model also demonstrated a satisfactory fit to the data (*χ*^*2*^ = 195.65, *p* < .001; CFI = .95; GFI = .95; RMSEA = .07). It accounted for 41% of the variance in the SPA factor. Figure 1 graphically illustrates the factor loadings and the associations between the six predictors and the SPA factor. Among the six predictor variables, perceived stress, gender, and BMI (normal vs. obesity) were significantly related to SPA scores (see Table 3). Notably, perceived stress was most predictive of the SPA scores (*β* = .47, *p* < .001), indicating that a 1 standard deviation (*SD*) increase in perceived stress was associated with a corresponding increase of .47 *SD* in SPA scores. In other words, higher levels of perceived stress were significantly linked to higher SPA scores.

**Table 3.** Standarlized Regression Weights(β) and Unstandardized Regression Weights (b), Standard Errors (S.E.), Critical Values (C.R.), and *p*-Values for the MIMIC Model.

	MIMIC model				
Covariates	*β*	*b*	*SE*	*CR*	*p*
Gender	.304	.494	.066	7.524	<.001
Age	−.004	−.001	.024	−.032	.974
Gender x Age	−.039	−.025	.033	−.752	.452
Perceived stress	.473	.058	.005	10.744	<.001
BMI (normal vs. Overweight)	−.002	−.006	.065	−.094	.925
BMI (normal vs. Obesity)	.147	.301	.079	3.818	<.001

Gender emerged as the second ranked predictor (*β* = .30, *p* < .001). On average, being female was linked to .30 *SD* higher SPA scores than being male, demonstrating that females were more likely to experience SPA compared to males. The next highly ranked predictor was BMI (normal vs. obesity), revealing that individuals with normal BMI values reported significantly lower SPA scores compared to those with obesity level BMI values (*β* = .15, *p* < .001). This implies that, on average, students with a normal BMI had .15 *SD* lower SPA scores than those with obesity level BMI values. However, the normal versus overweight comparison did not produce a significant predictive effect on the SPA score, indicating no discernible differences between students with a normal BMI and those with an overweight BMI. Finally, age and its interaction with gender was not a significant predictor of SPA scores.

## Discussion

We attempted to address notable gaps in existing research on SPA with this study. We employed a MIMIC model to explore simultaneously various possible predictors of SPA: gender, age, the interaction of age by gender, BMI category (normal vs. overweight; and normal vs. obesity), and perceived stress. Moreover, we focused on a neglected population, college kinesiology students. Our findings supported the adequacy of the revised 7-item one-factor SPAS for kinesiology students. Our MIMIC model also revealed an acceptable fit to the data and explained 41% of the variance in the SPA scores with our six predictors. In accordance with our hypothesis and previous research, our data supported a gender difference in self-reported SPA, and confirmed that females’ SPA levels tended to be significantly higher than males’ self-reported SPA, irrespective of age among these kinesiology students. Given that the measurement model of a revised one-factor SPAS exhibited complete gender invariance, the SPAS gender differences that emanated from the MIMIC model reflect gender disparities in SPA levels in this sample.

Several factors could have contributed to the observed higher levels of SPA among female kinesiology students compared to their male counterparts. First, the media, societal norms, and social expectations in the U.S. often place a greater emphasis on the physical appearance of women. The competitive nature of athletic environments in kinesiology could have exacerbated this gender imbalance in self-reported SPA. Second, compared to other academic fields, students in the kinesiology field may experience particular pressures regarding physical fitness and appearance. This emphasis within the kinesiology subculture could have contributed to heightened self-awareness and anxiety among female students, who might have perceived greater body image scrutiny or judgment. Given that gender disparities were found in both kinesiology and other academic fields (Chu et al., 2008; McLester et al., 2018). Future investigators might explore the potential interactive impact of gender by majors (i.e., kinesiology vs. non-kinesiology) on college students’ SPA.

In contrast to previous findings and our hypothesis, we did not observe age-related differences in SPA scores among kinesiology students. This may be due to our approach to classifying age variations. While many prior researchers categorized age into either two groups (e.g., Silva et al., 2022) or multiple groups (e.g., Hagger & Stevenson, 2010), potentially oversimplifying the intricate relationship between age and SPA. Rather, we leveraged the benefits of a large sample and the MIMIC model and treated age as a continuous variable, allowing for a more thorough exploration of its relationship to SPA levels. As per Osborne (2015), transforming a continuous variable into a categorical variable can introduce errors and lead to a significant loss of information, potentially resulting in spurious statistical significance. To confirm our speculation, we conducted a post-hoc independent *t* test to compare two age groups (18–20 years old, *n*_*1*_ = 377, *M* = 3.354. *SD* = .952; 21–24 years old, *n*_*2*_ = 216, *M* = 3.189. *SD* = 1.032), mirroring the analysis of age by classification groups that was used by Hagger and Stevenson (2010). These results revealed that the younger group had significantly higher SPA scores than the 21-24 group (*t*_*(591)*_ = 1.969, *p* = .049, Cohen’s *d* = .17). Thus, these two methods did produce variant results, and we believe the continuous variable approach to give the most accurate overall impression of age relationships to SPA.

We observed no significant interactive predictive effect on SPA of age by gender, meaning that the relationship between gender and SPA scores among kinesiology students was consistent across different ages. This finding aligns with observations of the age groups identified by Hagger and Stevenson (2010). The results from our two studies suggests that gender relationships to SPA scores may differ according to age only for students are less than 18 years old. Additionally, Hagger and Stevenson’s (2010) findings of age by gender effects on SPA scores did not consider or control for other potential predictors. In addition to age, gender, and their interaction), we additionally analyzed perceived stress, normal versus overweight, and normal versus obesity BMI values that contributed separate variance in predicting SPA scores.

Our data showed that students with normal BMI values displayed lower SPA scores compared to those with obesity BMI values, and this finding aligns with previous single variable research conducted with both adolescents (Mack et al., 2007) and adults (Ersöz et al., 2016; Hausenblas & Fallon, 2002; Russell, 2002; Zartaloudi et al., 2023), establishing BMI as a significant factor in determining the level of SPA across various age groups. However, as mentioned earlier, previous investigators mainly highlighted the overall contribution of BMI to SPA, while we examined the distinct impacts of different BMI categories and considered multiple variables simultaneously. Our data indicated that the relationship between BMI values and SPA primarily manifests in students with obesity rather than those with overweight. This finding has the potential to expand and improve our understanding of the relationships between BMI and SPA levels. Nonetheless, future investigators should explore this area further, particularly by comparing individuals with normal BMI to those classified as underweight, as individuals with an underweight BMI might also exhibit higher SPA levels. If this holds true, it would indicate a nonlinear relationship between BMI and SPA, challenging the previous argument or assumption that high BMI is associated with higher SPA levels based solely on the overall BMI (Sicilia et al., 2014; Zartaloudi et al., 2023).

Additionally, the noteworthy effects of BMI on SPA scores have been consistently observed across diverse studies. This underscores the importance of addressing body image concerns and promoting body positivity not only within the kinesiology student population but also among non-kinesiology students and among individuals of all age groups. Based on these findings, it is crucial for physical educators to implement strategies, create supportive environments, and develop interventions aimed at fostering a healthy body image and reducing SPA levels. To accomplish this, physical educators should prioritize creating opportunities for physical activities that emphasize enjoyment, skill development, and personal growth, rather than focusing on appearance-related outcomes. By doing so, physical educators can contribute to the establishment of a more inclusive and positive exercise environment for individuals of all backgrounds and body types.

Consistent with our expectations, our data revealed that higher levels of perceived stress were linked to elevated SPA scores. Surprisingly, perceived stress emerged as the strongest predictor of SPA scores among our six variables. This finding not only expands the current understanding of SPA but also highlights the significance of incorporating additional mental health-related variables such as mental toughness, depression, and mood in future SPA research. We expect that the inclusion of these additional mental health-related variables can provide researchers with a still more comprehensive understanding of the intricate interplay between psychological factors and SPA, ultimately contributing to the mitigation of SPA experiences among kinesiology students and others. Additionally, drawing from our findings, future college instructors and mental health professionals are encouraged to leverage the relationships between perceived stress and SPA levels in physical activity settings to help kinesiology students manage stress, improve body image, and reduce body image anxiety related to societal expectations.

### Limitations and Directions for Further Research

Four major limitations in this study merit attention. First, in our research design, an exclusive reliance on a self-reported questionnaires for data collection limited the objectivity of the data gathered. Second, our limited sample size restricted us to only two comparative categories of BMI values (i.e., normal vs. overweight; and normal vs. obesity). Future researchers should broaden the scope of BMI considerations by incorporating comparisons between normal and underweight, normal and overweight, normal and obesity, and normal and extreme obesity. This can be achieved through an enlarged sample size, with particular emphasis on increasing the representation of students with underweight and extreme obesity. Third, while our data showed that perceived stress was the most predictive of SPA scores, we cannot discern the directionality of the cause-effect relationship between perceived stress and SPA. Future researchers should explore this potentially reciprocal cause-effect relationship by employing a non-recursive model where there is a feedback loop between perceived stress and SPA constructs. Lastly, due to our limited sample size, we included only six independent variables in our MIMIC model. To enhance our understanding and expand the nomological networks regarding correlates of SPA, future researchers should examine additional potentially relevant variables. For example, considering that social anxiety and SPA relate to culture and ethnicity (Hofmann et al., 2010; Zwicker, 2021), future researchers should explore the impact of culture and ethnicity on kinesiology students’ SPA.

## Conclusions

In this study, we made three notable contributions to SPA research. First, this was the first study to focus on predictors of SPA among college kinesiology students. Given their focus on physical fitness and body-related aspects in academic and professional pursuits, kinesiology students may encounter specific SPA related to their field of study. These findings from a kinesiology student sample may deepen our understanding of different college students’ SPA experiences. Second, our use of a relatively novel MIMIC model overcame limitations of multiple regressions, ANOVAs, and MANOVAs, and allowed for a more comprehensive analysis of predictor variables. We recommend it as a primary statistical method for future SPA research. Lastly, we revealed that perceived stress was the largest predictor of SPA scores among our six proposed variables. This finding not only broadens the spectrum of the current nomological network regarding predictors of SPA but it also underscores the importance of incorporating additional mental health predictors (e.g., depression, mood, & mental toughness) in future SPA research.
